# A Twin Study of Mitochondrial DNA Polymorphisms Shows that Heteroplasmy at Multiple Sites Is Associated with mtDNA Variant 16093 but Not with Zygosity

**DOI:** 10.1371/journal.pone.0022332

**Published:** 2011-08-03

**Authors:** Toby Andrew, Cassandra D. Calloway, Sarah Stuart, Sang Hoon Lee, Raj Gill, Gail Clement, Philip Chowienczyk, Tim D. Spector, Ana M. Valdes

**Affiliations:** 1 Department of Twin Research and Genetic Epidemiology, King's College London, London, United Kingdom; 2 Children's Hospital Oakland Research Institute, Oakland, California, United States of America; 3 Department of Clinical Pharmacology, King's College London, London, United Kingdom; Ohio State University Medical Center, United States of America

## Abstract

The mitochondrial theory of ageing proposes that damage to mitochondria and diminished mitochondrial DNA (mtDNA) repair are major contributors to cellular dysfunction and age-related diseases. We investigate the prevalence of heteroplasmy in the mtDNA control region in buccal swab and blood derived samples for 178 women from the TwinsUK cohort (41 DZ pair 39 MZ pairs, 18 singletons, mean age 57.5 range 28–82) and its relationship to age, BMI and fasting insulin and glucose serum levels. The overall estimated prevalence of heteroplasmy for both tissues in the control region measured for 37 sites was 17%. The prevalence of heteroplasmy was higher among the older half of the study subjects than in the younger half (23% vs 10% p<0.03), primarily reflecting the increase in the prevalence of a heteroplasmic dinucleotide CA repeat in variable region II (VRII) with age. The VRII 523–524 heteroplasmic site (heteroplasmic in 25 subjects) was also associated with a decrease in BMI. In addition, concordance rates for common heteroplasmy were observed to be near complete for both dizygotic (DZ = 94%) and monozygotic twin pairs (MZ = 100%), consistent with previous reports that suggest variation in heteroplasmy rates between generations are determined by bottlenecks in maternal transmission of mitochondria. Differences in the prevalence of heteroplasmy were observed overall between samples derived from buccal swabs (19%) and blood (15%, p<0.04). These were particularly marked at position 16093 of hypervariable region I (HVI, 7% vs 0%, respectively, p<4×10^−11^). The presence of the C allele at position 16093 in blood was associated with the presence of heteroplasmy in buccal swabs at this position (p = 3.5×10^−14^) and also at VRII (p = 2×10^−4^) suggesting a possible predisposing role for this site in the accumulation of heteroplasmy. Our data indicate that BMI is potentially associated with control region heteroplasmy.

## Introduction

The mitochondrion represents a critical organelle for cell metabolism, signaling, energy production and apoptosis [1]. In mammals, mitochondria and mitochondrial DNA (mtDNA) are transmitted through the female germ line. Each diploid mammalian cell carries thousands of copies of mtDNA within it [2]. Due to the polyploid nature of the mitochondrial genome, wild-type and mutated mtDNA may coexist in individual organelles, or within a single cell or tissue; this condition is known as heteroplasmy. A cell or tissue can become heteroplasmic following a somatic or germ line point mutation at a specific site (point heteroplasmy) or an insertion/deletion mutation (length heteroplasmy).

In contrast to nuclear DNA, the mitochondrial genome is small (16,569 base pairs) and circular. Approximately 10% of the mitochondrial genome is non-coding, referred to as the control region, with two well characterised hyper-variable regions (HVI and HVII) and variable regions I (VRI) and II (VRII) [3]–[4].

Endogenous and exogenous agents frequently challenge the integrity of the mitochondrial genome. Specifically, reactive oxygen species (ROS) constitute a major endogenous source of mitochondrial DNA damage. It appears that ROS-induced mtDNA damage is more extensive and persists longer in comparison to nuclear DNA damage [5]. In addition, mtDNA also suffers damage from other toxic chemicals, to a greater extent than does nuclear DNA [6].

One of the prevalent theories of ageing, the mitochondrial theory, proposes that oxidative damage can eventually lead to dysfunctional or defective mitochondria (e.g. [7]–[8]). Chronic mitochondrial dysfunction is associated with a number of age-related degenerative diseases including Parkinson's disease, Alzheimer's disease and age related macular degeneration (AMD) [9]–[11].

A recent study of common mtDNA heteroplasmy (heteroplasmy assay sensitivity>0.05) examined 625 mother–child pairs and observed that heteroplasmy was inherited approximately 30% of the time, but more typically would occur de novo in offspring or, conversely, be present in mothers but would not be observed in their children (70%) [12]. Moreover, the frequency of heteroplasmic variants has been reported to vary between different tissues in the same individual and cancer cells harbour homoplasmic and heteroplasmic mtDNA mutations not present in blood from the same individual [13].

In this study we investigate the correlation of heteroplasmy in the mtDNA control region with age, BMI and fasting insulin and glucose serum levels in two different tissues from 178 women. In addition, by using a classical twin design, we investigate the influence of shared zygosity upon heteroplasmy concordance rates. Finally, we show that mtDNA polymorphisms at 16093 are strongly associated with the prevalence of heteroplasmy at a dinucleotide repeat site.

## Methods

### Study subjects

The study participants were 178 female Caucasian monozygotic (MZ) and dizygotic (DZ) twins from the TwinsUK adult twin registry, collected to study the heritability and genetics of age-related diseases (www.twinsuk.ac.uk). These unselected twins were recruited from the general population through national media campaigns in the UK and shown to be comparable to age-matched population singletons in terms of disease-related and lifestyle characteristics [14]. Weight was measured using scales with subjects dressed in light clothes and height was measured using a stadiometer. Fasting insulin and glucose levels were measured for the twin cohort using the same methods as previously described [15]. The study was approved by St Thomas' Hospital Research Ethics Committee and all twins provided informed written consent. In total 39 MZ and 41 DZ pairs were included in the study in addition to 18 individuals whose co-twin did not participate.

### DNA extraction

Total genomic DNA was previously extracted from blood from 165 of the donors using standard protocols. In addition, a buccal sample was collected from all subjects using the isohelix buccal swab (Cell Projects Ltd, Kent UK) and stored in a stabilizing solution at −20C until the DNA isolation was performed. DNA from the buccal sample was isolated using the Isohelix DNA Isolation Kit (Cell Projects Ltd, Kent UK) following the manufacturer's procedure. The lyophilized DNA was resuspended in 150 uL of the provided TE buffer. The DNA from buccal and blood samples were quantified using a Nanodrop Spectrophotometer.

### mtDNA Amplification

DNA from buccal and blood samples were amplified using a duplex PCR to target the entire non-coding region of the mitochondrial genome. Approximately 1–10 ng of DNA extracted from buccal samples and 1–2 ng of DNA extracted from blood samples were amplified in 50 µL reactions containing 12 mM Tris-HCl (pH 8.3), 60 mM KCl, 2.4 mM MgCl_2_, 0.2 mM each dNTP, 0.25 U/µL AmpliTaq Gold DNA polymerase (Applied Biosystems, Foster City, CA), and 0.3 µM of each primer. PCR cycling conditions were as follows: one polymerase activation cycle at 94°C for 14 minutes; 34 or 38 cycles of amplification, consisting of denaturation at 92°C for 15 s, annealing at 59°C for 30 s, and extension at 72°C for 30 s; and one final extension cycle at 72°C for 10 minutes. Two pairs of biotinylated primers were used to simultaneously amplify the HVI-VRI and HVII-VRII regions of the human mitochondrial genome. Primers F15975-93 and R12-16526 were used to amplify the HVI and VRI regions (∼607 bp) and F15-34 and R568-49 were used to amplify the HVII and VRII regions (∼554 bp). Primer numbers correspond to the revised Cambridge Reference Sequence (rCRS, NC_012920) [16]. A subset of the samples were resolved by gel electrophoresis using a 3% Agarose LE gel in 1× TBE buffer containing 0.5 mg/mL ethidium bromide at 125 V for approximately 1 hour. Samples were compared to a low mass DNA ladder to determine amplification success and to estimate PCR product yield.

### mtDNA Typing

A probe panel consisting of 65 probes striped in 63 lines targeting 37 polymorphisms in the non-coding control region of the mitochondrial genome was used to characterize heteroplasmy for this study. The polymorphic positions targeted with this probe panel include 14 within HVI, 13 within HVII, four within VRI and six within VRII and are listed in **Supplementary Table S1**. This probe panel also includes 33 probes targeting 18 sites from the LINEAR ARRAY™ Mitochondrial DNA HVI/II Region-Sequence Typing Kit (Roche Applied Science, Indianapolis, IN, USA) as well as probes targeting non-coding polymorphisms from a next generation version (http://www.ncjrs.gov/App/Publications/abstract.aspx?ID=232781) Polymorphic sites targeted are among the most mutable and in many cases, reported heteroplasmic hotspots (e.g. 16093, 152, 189, 414). A set of probes targeting the 414 heteroplasmic hotspot were also included in this panel. All probes were previously optimized, tested for specificity and validated.

Samples were typed following the manufacturers instructions for the LINEAR ARRAY™ Mitochondrial DNA HVI/II Region-Sequence Typing Kit (Roche Applied Science, Indianapolis, IN, USA) with one modification. For all typing steps the solution volume was increased from three mL to four mL keeping all reagent concentrations constant. Following typing the probe signal intensities and patterns were determined visually using a probe panel template. Four different types of probe binding patterns may be observed in each probe region. A positive signal is observed when a sequence hybridizes to a complementary probe. A sequence with a destabilizing or a highly destabilizing mismatch within the probe binding region will result in a weak or no hybridization signal. Two probe signals are expected within a region in samples where heteroplasmy is observed. Similar probe panels have been used to characterize heteroplasmy and were shown to be sensitive enough to detect heteroplasmy where the minor component is approximately 5–10% dependent on the sample concentration and the probe region [17]. All probe calls were cross checked for accuracy by two scientists. All samples that weakly amplified or were successfully typed but showed discrepancies or evidence of heteroplasmy were re-amplified and retyped. Sample input and in a few cases, PCR cycle number, were increased for samples exhibiting weak amplification.

### Dye terminator confirmation sequencing

Confirmation for a subset of samples from both buccal swabs and blood was performed by PCR amplification and conventional Sanger dye terminator sequencing, with the ABI Prism 310 Genetic Analyzer following the recommended instructions. Sequencing data were analyzed by SEQUENCHER software packages (Gene Codes Corporation).

### Statistical Analysis

The tests for association between age, BMI, fasting insulin and glucose serum levels and the presence of heteroplasmy were carried out using linear (logistic and ordinary least squares) regression with a robust estimate of the standard error for the beta coefficient calculated by clustering over twin pairs (individual sample analysis) and individuals (both tissue samples included in the statistical analysis). The genetic association between the fixed allelic status at HVI site 16093 in blood and heteroplasmy at the same and other sites were also tested using robust logistic regression. For regression analyses, the likelihood ratio test (LRT) statistic p-value is presented to assess the overall model fit. Regression beta coefficient p-values are presented for multiple regression analyses to assess whether variables are independently associated with site heteroplasmy or not. For logistic regression, the pseudo-R^2^ model-fit statistic is analogous (but not directly comparable) to the ordinary least squares regression R^2^ statistic, known as the coefficient of determination. While R^2^ can be interpreted as the proportion of variance explained by the model, pseudo-R^2^ is loosely interpreted as the proportion of variation in risk liability explained by the model [18].

### Twin concordance

The classical twin model contrasts the degree of concordance within monozygotic (MZ) twins with that of dizygotic (DZ) twins based upon the premise that MZ share 100% of their inherited nuclear (and mitochondrial) DNA, while DZ twins like fraternal siblings, on average only share half their nuclear DNA. Genetic inferences can be drawn if MZ concordance exceeds DZ concordance rates on the assumption that the phenotypic intra-class correlation is not confounded by zygosity status [19]. In the case of heteroplasmy however, MZ twins not only share the same inherited nuclear DNA, but also as a consequence of being derived from the same zygote, share exactly the same inherited maternal cytoplasm and mitochondrial polyploid cell composition. By contrast, DZ twins will on average share half their inherited nuclear DNA, but the mitochondrial cell composition at fertilization, while similar, will not be exactly the same, since each twin originates from two distinct zygotes with potentially distinct bottleneck events during gametogenesis [20]. Hence the contrast in heteroplasmy concordance rates between MZ and DZ twins will not provide a standard heritability estimate, but an estimate of “nuclear heritability plus shared bottleneck” effects.

For twin similarity in dichotomous traits, case-wise concordance is the probability of observing heteroplasmy in a twin conditional upon their co-twin being heteroplasmic at the same site (and for the same heteroplasmy threshold). Case-wise concordance (C) is a measure of individual risk and is directly comparable to the commonly used genetic epidemiological measure of λ_R_ (the probability of being affected conditional upon an affected relative divided by the population disease prevalence), with λ_R_ estimated from twin data as C_MZ_/C_DZ_. Heteroplasmy case-wise concordance rates for MZ and DZ twin pairs were computed using the method described by Witte et al [21]. To assess the statistical significance of the difference between MZ and DZ casewise concordance rates and to determine the confidence intervals of the casewise concordance estimates 10,000,000 bootstrap iterations were performed. All analyses were carried out using the STATA package [18].

## Results

**Figure 1:**
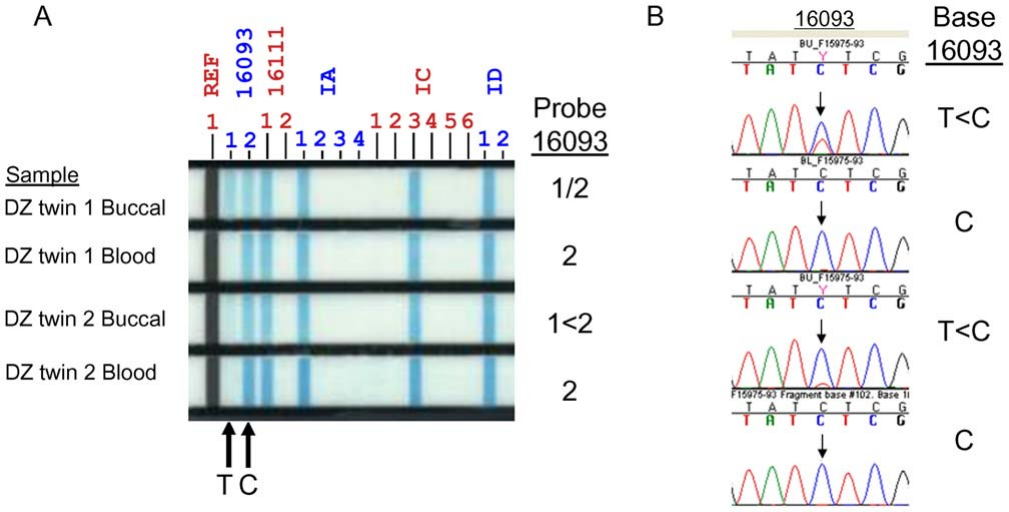
Heteroplasmy at position 16093 detected in buccal but not blood samples in a dizygotic twin pair aged 63 at the time of the study by A) Linear array assay and B) confirmed by dye terminator sequencing. Two probes signals, 16093 1 and 16093 2, were detected by linear array analysis in the buccal samples but not in the blood of this twin pair, corresponding to 16093 T and C. The relative amounts of the 16093 T and C differed between the twins in the buccal and are reflected both by the intensity of the probe signals (A) as well as the peak height ratio in the sequence chromatogram (B).

**Figure 2:**
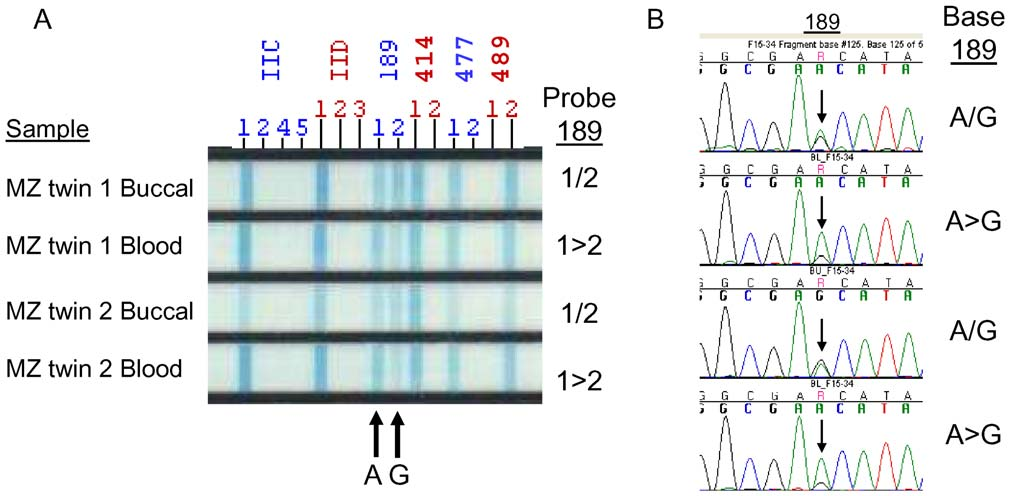
Heteroplasmy at position 189 detected in buccal and blood samples in a monozygotic twin pair aged 60 at the time of the study by A) Linear array assay and B) confirmed by dye terminator sequencing. Two probe signals were observed within the 189 region corresponding to 189 A and G sequence in both the buccal and blood samples of this twin pair. Similar probe signal intensities and peak heights were observed in the buccal samples. The probe signal and sequence peak corresponding to 189 A was greater in the blood samples compared to the 189.

The measurement of heteroplasmy from the linear array was found to be reliable compared with conventional sequencing and is illustrated for two previously reported heteroplamsy hotspots, position 16093 (**Figure 1**) and position 189 (**Figure 2**). Five of the 37 sites tested were observed to be heteroplasmic.

The mean age, BMI, fasting insulin and glucose serum levels of study subjects and the length and point heteroplasmy counts in buccal swab samples are shown in **Table 1**. The overall prevalence of heteroplasmy in both tissues for the polymorphic sites was estimated to be 17%, with prevalence of each of these sites in the 178 buccal swab samples and 165 blood samples is shown in **Table 2**.

**Table 1:** Mean age, body mass index (BMI) and insulin and glucose fasting serum levels for heteroplasmic and non-heteroplasmic individuals (heteroplasmy measured in buccals swabs).

			Age (years)		BMI (kg/m^2^)		Insulin (pmol/l)		Glucose (mmol/l)	
Heteroplasmy type:	Heteroplasmic	n	Mean	SD	Mean	SD	Mean	SD	Mean	SD
Any (control region)	N	145	57	9.1	26	4.8	59	44.6	5.1	0.58
	Y	33	57	8.9	26	6.7	53	39.8	5.0	0.56
Point: HVI (16093)	N	165	57	9.0	26	4.8	59	45.0	5.1	0.59
	Y	13	58	9.2	28	8.9	45	22.5	4.9	0.37
Point: HVII (64 & 189)	N	174	57	9.0	26	5.2	57	42.4	5.1	0.56
	Y	4	51	10.3	31	2.7	155	8.5	6.3	0.35
Length: VRII (523–524)	N	153	57	9.2	26	4.8	59	44.1	5.1	0.58
	Y	25	60	7.1	26	7.5	55	43.1	5.0	0.59

**Table 2:** Prevalence of control region heteroplasmy in mtDNA for buccal swab and blood tissue samples taken from the same individuals.

Heteroplasmy		Buccal samples (n = 178)			Blood samples (n = 165)			
		y	n	Prevalence	y	n	Prevalence	p-value
**All sites**		33	145	19%	24	141	15%	0.04
**HVI**	all	14	165	8%	0	165	0	1.7E-11
	16093	13	165	7%	0	165	0%	3.6E-11
	16304 to 16320	1	177	1%	0	165	0	-
	16111	0	178	0	0	165	0	-
	16124 to 16129	0	178	0	0	165	0	-
	16362	0	178	0	0	165	0	-
	16264 to 16278	0	178	0	0	165	0	-
**HVII**	all	4	174	2%	4	161	2%	0.19
	64	2	176	1%	2	163	1%	0.34
	189	2	176	1%	2	163	1%	0.34
	72	0	178	0	0	165	0	-
	73	0	178	0	0	165	0	-
	200	0	178	0	0	165	0	-
	247	0	178	0	0	165	0	-
**VRI**	16519	0	178	0%	0	165	0%	-
**VRII**	523 to 524	25	153	14%	22	143	13%	0.49
	414	0	178	0	0	165	0	-
	477 to 482	0	178	0	0	165	0	-
	489 to 493	0	178	0	0	165	0	-
	523 to 524	25	153	14%	22	143	13%	0.49

All variants are base substitutions (at a single point or within a specified sequence range), except VRII 523 to 524, which is an insertion-deletion. p-values are reported for a 1df robust logistic regression (standard error of the beta coefficient calculated clustered by twin pair) contrasting prevalence of heteroplasmy between buccal and blood samples. HVI = hyper-variable region I; HVII = hyper-variable region II; VRI = variable region I; VRII = variable region II.

We compared the rates of heteroplasmy between buccal swab data and blood data from the same individuals and observed no difference in prevalence of heteroplasmy at the VRI, VRII or HVII sites between blood and buccal samples. However, the HVI 16093 point heteroplasmy was observed to be heteroplasmic in buccal swabs, but not blood (**Table 2**). Heteroplasmy at hypervariable region I (HVI) position 16093 was observed to be 7% in buccal swab samples, but 0% in blood samples with difference in prevalence statistically significant at p = 4×10^−11^. No significant difference was seen between blood and buccal swabs at any of the other sites (**Table 2**).

For the study assay detection limit (∼5–10%) used to reliably call heteroplasmy, we observed no significant difference in the mean age of heteroplasmic and non-heteroplasmic individuals. However, we did observe that the overall prevalence of heteroplasmy was two-fold higher in the older half of the study subjects compared to the younger half for buccal swabs, blood and in both tissues considered jointly (p = 0.03, **Table 3**). When we inspected difference in prevalence by site, we observed that there was marginal statistical evidence (p<0.05) for correlation between age and heteroplasmy at insertion/deletion in positions 523–524 for both types of tissue (**Table 3**) with prevalence of heteroplasmy at this site being higher among the older individuals. There was no difference in age at the three other heteroplasmic control region sites.

**Table 3:** Heteroplasmic association with age and body mass index (BMI).

Heteroplasmy		Buccal	(n = 178)				Blood	(n = 165)			Pooled		(n = 343)		
			*Prevalence:*					*Prevalence:*					*Prevalence:*		
	OR	p	Lower	Upper	p	OR	p	Lower	Upper	p	OR	p	Lower	Upper	p
**All sites**															
Age	1.40	0.47	12%	26%	0.07	1.21	0.91	9%	20%	0.12	1.25	0.86	10%	23%	0.03
BMI	0.68	0.10	23%	14%	0.13	0.70	0.25	17%	12%	0.22	0.69	0.17	20%	13%	0.19
Age (adj. BMI)	1.28	0.35				1.31	1.00				1.00	0.926			
BMI (adj. Age)	0.65	0.10			0.09	0.66	0.91			0.21	0.90	0.181			0.08
**HVI 16093**															
Age	1.50	0.48	4%	10%	0.33	-	-	0%	0%	-	-	-	2%	5%	-
BMI	0.66	0.03	9%	6%	0.11	-	-	0%	0%	-	-	-	5%	3%	-
Age (adj. BMI)	1.38	0.80				-	-				-	-			
BMI (adj. Age)	0.59	0.02			0.26	-	-			-	-	-			-
**HVII 64 & 189**															
Age	0.67	0.30	2%	2%	0.87	0.64	0.25	2%	2%	0.82	0.66	0.28	2%	2%	0.99
BMI	5.35	0.0003	0%	4%	-	5.09	0.0004	0%	5%	-	5.22	0.0003	0%	5%	-
Age (adj. BMI)	0.62	0.235				0.58	0.20				0.60	0.22			
BMI (adj. Age)	5.42	0.001			-	5.29	0.001			-	5.35	0.001			-
**VRII 523 to 524**															
Age	1.08	0.02	5%	23%	0.01	1.42	0.19	6%	21%	0.03	1.52	0.12	6%	22%	0.02
BMI	0.59	0.08	18%	10%	0.03	0.61	0.14	17%	10%	0.05	0.61	0.12	17%	10%	0.04
Age (adj. BMI)	1.84	0.02				1.62	0.07				1.73	0.04			
BMI (adj. Age)	0.53	0.05			0.002	0.54	0.08			0.01	0.53	0.07			0.004

Odds ratios and corresponding p-values are reported using robust logistic regression. Heteroplasmy status is regressed upon age and BMI with univariate analyses odds ratios (OR) and model-fit likelihood ratio test (LRT) p-values presented. Adjusted odds ratios (OR) and p-values are also presented for the corresponding multiple regressions for age+BMI. Odds ratios are reported as quartiles of age and BMI (first column), with the model fit statistics (p-value) reported using the more informative continuous measures of age and BMI (second column). The p-value column reported with prevalence corresponds to the regression beta-coefficient p-values for age and BMI and multiple regression model-fit LRT p-value, respectively, with heteroplasmy regressed upon dichotomised age and BMI (upper and lower quartiles). Four individuals are observed with HVII point mutations (all 4 individuals have a BMI over 28). Twenty-two individuals are observed with VRII insertion-deletions in blood and buccal samples (between base pairs 523 to 524) and an additional 3 mutations were observed at this site in buccal samples only.

Heteroplasmic status at the same VRII position (523–524) also appeared to be more strongly associated with age when considered in conjunction with BMI using multiple regression. This was true for buccal, blood and combined tissue analyses (p = 0.004, **Table 3**) with prevalence of heteroplasmy increasing between the lower and upper median quantiles of age (6% vs 22%), but decreasing with BMI median quantiles (17% versus 10%). For combined tissue analyses, the adjusted odds ratio (OR) for heteroplasmy at position 523–524 was 1.7 for each quartile increment in age and OR = 0.5 per quartile increment in BMI (p = 0.03, **Table 3**), while for the corresponding estimates for dichotomised age and BMI (not shown) were OR = 5.4 and OR = 0.3, respectively (p = 0.004; pseudo-R^2^ = 0.11. The latter indicates that BMI and age jointly account for approximately 11% of the variance in heteroplasmy liability at VRII 523–524).

BMI was also associated with heteroplasmy at HVII, while age was not. Although the numbers of individuals observed to be heteroplasmic at HVII (sites 64 and 189) were low (n = 4), these individuals showed a large mean increase in BMI (4.3 kg/m^2^, p = 0.001) and fasting insulin (98.1 pmol/l, p = 5×10^−6^, **Table 4**) and glucose (1.2 mmol/l, p = 4×10^−5^, **Table 4**) serum levels. The prevalence of heteroplasmy at this site was zero for the lower quartile and approximately 3% in the upper quartile for all three traits (**Table 4**). All four individuals were over-weight (BMI 28–34) and showed signs of insulin resistance (as measured by minimum fasting insulin>148 pmol/l and glucose>6 mmol/l), but their mean age was no different to non-heteroplasmic individuals at HVII.

**Table 4:** Heteroplasmic association with insulin (pmol/l) and glucose (mmol/l) fasting serum levels.

Heteroplasmy	Buccal (n = 131)				Blood (n = 119)				Pooled samples (n = 343)			
			*Prevalence:*				*Prevalence:*				*Prevalence:*	
	β	p	Lower	Upper	β	p	Lower	Upper	β	p	Lower	Upper
**All sites**												
Insulin	−6.2	0.65	19%	11%	−3.5	0.84	15%	8%	−5.0	0.74	17%	10%
Glucose	−0.1	0.54	20%	12%	−0.1	0.78	14%	11%	−0.1	0.65	17%	11%
IGR	−1.4	0.53	17%	13%	−1.0	0.69	15%	8%	−1.2	0.60	16%	11%
IGR (adj. for age+BMI)	−1.0	0.62			−1.1	0.65			−1.1	0.62		
**HVI 16093**												
Insulin	−14.1	0.17	8%	6%	-	-	0%	0%	−13.7	0.17	4%	3%
Glucose	−0.2	0.16	10%	3%	-	-	0%	0%	−0.2	0.16	5%	2%
IGR	−2.3	0.21	6%	8%	-	-	0%	0%	−2.2	0.21	3%	4%
IGR (adj. for age+BMI)	−2.9	0.20			-	-			−2.9	0.19		
**HVII 64 & 189**												
Insulin	98.1	0.001	0%	3%	98.1	0.002	0%	3%	98.1	5.E-06	0%	3%
Glucose	1.2	0.004	0%	3%	1.2	0.005	0%	4%	1.2	4.E-05	0%	3%
IGR	13.8	0.01	0%	3%	13.8	0.017	0%	3%	13.8	0.001	0%	3%
IGR (adj. for age+BMI)	11.3	0.04			11.2	0.04			11.2	0.003		
**VRII 523 to 524**												
Insulin	−4.1	0.79	16%	10%	−3.5	0.84	15%	8%	−3.8	0.82	15%	9%
Glucose	−0.1	0.74	15%	12%	−0.1	0.78	14%	11%	−0.1	0.76	15%	11%
IGR	−1.1	0.64	16%	9%	−1.0	0.69	15%	8%	−1.1	0.66	15%	9%
IGR (adj. for age+BMI)	−1.0	0.67			−1.1	0.65			−1.1	0.65		

Fasting glucose, insulin and insulin∶glucose ratio (IGR) are regressed upon heteroplasmic status (univariate) and heteroplasmic status, age and BMI (multiple regression). β = regression beta coefficient; p = regression coefficient p-value. For example, these analyses indicate that the four individuals (2 concordant monozygotic twin pairs) that show heteroplasmy at HVII for both buccal and blood samples, have a mean raised insulin and glucose serum levels of 98.1 pmol/l and 1.2 mmol/l, respectively. Prevalence of heteroplasmy is reported for the trait Lower (insulin 9–47 pmol/l; glucose 4–5 mmol/l; IGR 0.02–0.11) and Upper quantiles (insulin 48–246 pmol/l; glucose 5–7.3 mmol/l; IGR 0.11–0.49).

### Association of position 16093 with heteroplasmy

In this study we observe blood data, which are not heteroplasmic at position 16093, and buccal swab data, which are heteroplasmic at this site. We find that the presence of the C allele at 16093 in blood mtDNA is strongly associated with the presence of heteroplasmy in buccal samples at the same HVI site (**Table 5**). In addition the C allele at HVI 16093 in blood is also associated with heteroplasmy at VRII for both buccal and blood samples (**Table 5**). The relative risk of being heteroplasmic at VRII 523–524 for those with a C allele at 16093 is approximately six times those with a T allele (**Table 5**).

**Table 5:** Genetic association between the fixed minor allele at HVI site 16093 observed in blood and heteroplasmy in both tissue samples (buccal swab and blood).

Heteroplasmy:		At any site				HVI (16093)			HVII (64 & 189)			VRII (523–524)			
	*Tissue*	N(%)	Y(%)	OR(95% CI)	p	N(%)	Y(%)	p	N(%)	Y(%)	p	N(%)	Y(%)	OR(95% CI)	p
Allele at 16093 in blood	**Buccal**														
T		134(88)	19(12)	-	4.6E-09	153(100)	0(0)	3.5E-14	149(97)	4(3)	0.50	136(89)	17(11)	16.0(3.0–79.0)	0.0002
C		0(0)	9(100)			1(11)	8(89)		9(100)	0(0)		3(33)	6(67)		
	**Blood**														
T		134(88)	19(12)	11.2(3.0–44.0)	0.0006	153(100)	0(0)		149(97)	4(3)	0.47	136(89)	17(11)	12.8(3.0–50.0)	3.1E-04
C		4(40)	6(60)			10(100)	0(0)		10(100)	0(0)		4(40)	6(60)		

Odds ratios and their corresponding p-values from logistic regression analyses are presented for those contingency tables with no zero count cells; otherwise p-values corresponding to Pearson chi-square statistics are presented.

*Twin concordance*: MZ twins were completely (100%) concordant for total heteroplasmy status in the control region. The DZ case-wise concordance was found to be 94.1% (95% CI 83%–106%), with only one pair discordant for heteroplasmy in the control region. We used bootstrap methods to empirically assess whether this slight difference in concordance between MZ and DZ twins was statistically significant and observed the difference not to differ significantly from zero (5.9%, 95% CI: −7.3%–19.1% p<0.38).

## Discussion

For this study we observed mtDNA heteroplasmy to be only weakly associated with age at one insertion/deletion site. The explanation for this is likely to be that the common heteroplasmy we detected for these samples (probe sensitivity > = ∼5–10%) were largely inherited rather than somatic. It may also be the case that even if somatic mutations are associated with human chronological age [12], [22] the accumulation of somatic mitochondrial mutations do not promote ageing. For example, mice heterozygous for the proofreading- deficient polymerase g have been shown to accumulate high levels of somatic mtDNA mutations at a 500-fold higher mutation burden than normal mice. Yet these heterozygote mutants do not display features of rapidly accelerated ageing suggesting that mitochondrial mutations may not promote ageing or limit the lifespan of wild-type mice [23]. While our data suggest that common control region heteroplasmy are either not or only weakly associated with age, this does not preclude stronger association between age and the accumulation of somatic mutations, which have largely gone undetected in this study. Other studies have reported association with chronological age, but they have used different tissues such as muscle [22].

In our study we find that 16093 is heteroplasmic in buccal swabs, but not blood samples. Differences in the rate of heteroplasmy for this variant between tissues of the same individual have been previously reported. He and co-workers [13] studied position 16093 in 10 different tissue types (but not blood) from the same person and identified the locus to be heteroplasmic in all 10 tissues with the frequency of the C allele varying within this individual from 7.4% in skeletal muscle to 90.9% in kidney. Therefore both these published data and ours indicate that heteroplasmy at this position is tissue dependent.

Our data are also in agreement with other studies which found a very high prevalence of heteroplasmy at position 16093. Irwin et al [24] observed the highest rate of heteroplasmy at position 16093 in a study of 19 populations and noted that in the majority of heteroplasmy cases (70%) the tissue had the C allele as the predominant base despite the fact that the C allele is the minor allele in the population with a frequency of around 6%. Tully and co-workers [25] had already suggested that some form of positive selection for the T allele may be acting at the same time there is a strong propensity for mutation at this site. When comparing two types of tissues, blood and buccal swab samples, we find that the presence of the C allele at this site in blood, results in a higher prevalence of heteroplasmy in buccal samples at the same site and is associated with increased heteroplasmy at VRII 523–524 for buccal and blood samples.

While age was only marginally associated with heteroplasmy at VRII 523–524, in conjunction with BMI it was more strongly associated with heteroplasmy at this insertion-deletion site. BMI was also associated with point mutation heteroplasmy at HVII (positions 64 and 189). It is likely that the causes of phenotypic association with heteroplasmy at these sites will differ by type of mitochondrial DNA mutation. We hypothesize that the positive association between the point mutation heteroplasmy at HVII and BMI and insulin resistance might reflect the accumulation of somatic mutations driven by weight-related health status, while the negative association between BMI and VRII 523–524 might be causally driven by a accumulation of length polymorphism heteroplasmy resulting in a loss of adiposity with age. Increased accumulation of heteroplasmy at VRII 523–524 is associated with and perhaps even driven by the presence of the C allele polymorphism at 16093. Why the length polymorphism heteroplasmy might be associated with lower BMI is not clear. However, similar results have also been observed for oxidative phosphorylation dysfunction, where inherited deleterious mutations lead to the majority of patients with multisystemic oxidative phosphorylation diseases being lean rather than obese [26]. We speculate that this may be due to mitochondrial dysfunction in relation to thermogenesis regulation, or energy wasting or increased fuel consumption to maintain ATP levels.

We also find that rates of heteroplasmy are significantly different between the two types of tissue studied, which is consistent with the growing recognition of the widespread differential somatic heteroplasmy rates between different organs and tissues [13], [27]. Thus, it is possible that in other tissues, such as the retina or the arterial wall, the prevalence of heteroplasmy might be closely related to cell duress. Unlike previous reports, we find the largest occurrence of common heteroplasmy in VRII and not in one of the hypervariable regions, which is likely to reflect the higher threshold used by the linear array assay to declare heteroplasmy compared to the sequencing technology used by other authors [13], [27]. In addition in the present study we only analysed a limited number of sites unlike other studies which have sequenced the whole control region.

We did not observe an association between age and heteroplasmy at position 189 for blood and buccal samples, which is in agreement with previous reports that only observed association at this position from mtDNA derived from muscle cells [22], [28]. Calloway and co-workers [28] failed to see an association between age and heteroplasmy at 189 in blood using the same line array technology used here. Theves et al [22] saw only a weak association with age in buccal cells using a PNA/real-time PCR method, and this association was due to the inclusion of very young subjects (including an infant) which is not true for the present study, where the youngest subject was aged 28. Thus, the fact that we do not find a significant relationship between age and heteroplasmy in buccal and blood cells at position 189 is in line with those reports.

Our data, in conjunction with previous published studies [13], [22], [24], [27], suggests that mtDNA variations are not only a pre-condition for mitochondrial heteroplasmy at the same site, but also that the same hyper-variable sites such as 16093 may drive the variability in heteroplasmy rates at other sites and between tissues. Furthermore, we did not observe evidence for a shared zygote and nuclear genetic contribution to heteroplasmy, as witnessed by the same high concordance rates in DZ and MZ twins.

We note several limitations to this pilot twin study, the main one being the sample size is relatively small (n = 178) compared with other studies and that the findings are subject to replication. However, our data are largely consistent with previous studies and in particular the C allele at 16093 associated with increased prevalence of heteroplasmy is in agreement with the largest study of mtDNA control region heteroplasmy published to date [24], [27] and supports the validity of our data.

In conclusion, common heteroplasmy in the mtDNA control region derived from peripheral tissues are only weakly correlated with age, but appear to be more consistently associated with BMI for at least one control region site. The association between the C allele at position 16093 and heteroplasmy at VRII suggests that mitochondrial DNA polymorphisms may play a predisposing role in the accumulation of heteroplasmy.

## Supporting Information

Table S1 mtDNA sequence variation targeted by the linear array used in the present study. Base positions correspond the rCRS sequence [16]. The probe number for the 33 probes included from the Linear Array HVI/HVII Region Sequence Typing Kit are underlined.
(XLS)
